# Proof of concept of fully automated adaptive workflow for head and neck radiotherapy treatments with a conventional linear accelerator

**DOI:** 10.3389/fonc.2025.1382537

**Published:** 2025-01-23

**Authors:** Gaia Muti, Marco M. J. Felisi, Angelo F. Monti, Chiara Carsana, Roberto Pellegrini, Edoardo Salmeri, Mauro Palazzi, Paola E. Colombo

**Affiliations:** ^1^ Medical Physics Department, Azienda Socio Sanitaria Territoriale Grande Ospedale Metropolitano (ASST GOM) Niguarda, Milano, Italy; ^2^ Radioteraphy Department, ASST GOM Niguarda, Milano, Italy; ^3^ Elekta AB, Medical Affairs, Stockholm, Sweden

**Keywords:** auto-planning, MCO, online adaptive radiotherapy, offline adaptive radiotherapy, CBCT, adapt to shape, fluence to position

## Abstract

**Introduction:**

The objective of this study is to evaluate the performance of an automatic workflow for head-and-neck (H&N) radiotherapy using a multi-atlas based auto-contouring software and an a-priori multicriteria plan optimization algorithm and implement an adaptive online approach with CBCT images. Two different modalities are investigated, the fluence-to-position (FTP) and the adapt-to-shape (ATS) approach.

**Materials and methods:**

Nine patients are used for the multi-atlas database. The organs at risk (OARs) of the H&N district and five additional structures (air, fat, tissue, bone and patient’s exterior) subsequently used for the creation of the synthetic CT are auto-contoured with the Elekta ADMIRE^®^ software. The mCycle algorithm is used for the a-priori multicriteria plan calculation. A total of twenty H&N patients are selected for this step. The automatic plans are compared to manual VMAT plans by assessing differences in planning time, dose delivered to targets and OARs, and calculating the plan quality indexes (PQIs). Two patients are chosen for the retrospective CBCT adaptive online feasibility analysis. To assess the differences for the two adaptive modalities, the clinical goals for targets and OARs and the number of passed constraints are explored. An analysis of the timing for the different steps is carried out to assess its clinical applicability.

**Result:**

The dice of the five HU layer structures range between 0.66 and 0.99. The mCycle auto-planning significantly reduces planning time, from 2 hours to 10 minutes. The radiotherapist deems all plans clinically acceptable, and in the majority of cases the automatic plan is the preference choice. The automatic plans enhance OARs sparing and preserve a good target coverage, this is also confirmed by the PQIs result. Comparing FTP and ATS modes in adaptive radiotherapy, ATS exhibits superior outcomes, mostly in the target coverage. In the FTP techniques target coverage is inadequate and statistically different from the accepted values. In the ATS the results align with the initial approved values. Using the ATS mode the planning time takes around 14 minutes and approximately 20 minutes for the entire treatment.

**Conclusion:**

This study contributes to the advancement of automatic and adaptive radiotherapy, demonstrating the potential of an automated workflow in H&N treatments.

## 1 Introduction

Head and neck cancer is a challenging site to treat in terms of contour definition, planning technique and anatomical changes between sessions. Anatomical changes may occur from as early as the first irradiation sessions. Inter-fraction changes, that include shrinkage of the tumor and/or normal tissue, result in target movement in different positions relative to other structures (1).

Adaptive radiation therapy (ART) is a process to control anatomical variations over the treatment course. This provides a day-by-day representation of the patient’s anatomy to better delineate the target and the OARs volumes (2). ART can be performed online or offline: both accommodate anatomical changes during treatment, but differ in their implementation and timing of treatment plan adaptations. Offline ART relies on periodic, usually CT, imaging sessions, which are separated from the actual treatment. It focuses on adapting the treatment plan periodically for future sessions. Online ART involves the observation of the patient’s anatomy using imaging techniques, such as CBCT or MRI, and then it assesses the anatomical or position changes before the treatment. In this way the treatment plan is continually updated on a daily basis to account for the current anatomical configuration. Online ART aims to enhance treatment accuracy by reducing setup uncertainties and improving target localization. In recent years, the rise of MRI-LINACs and the resulting MRI image-guided radiotherapy (MRgRT) (3) has renewed the interest in the field of adaptive online treatment, which also has led to the investigation of ART with CBCT images to promote its applicability on conventional linear accelerators.

The CBCT online ART workflow begins with a CBCT acquisition, creating new reference images. PTVs and OARs can be propagated from the planning CT onto the current CBCT. For the CBCT planning, their inaccuracy in Hounsfield units (HU) and electron densities could induce a non-negligible dose error (4). For this reason the use of a synthetic CT (sCT) in CBCT planning is an essential point. The densities of each volume are calculated from the initial planning CT and subsequently assigned to the contours propagated on the CBCT, resulting in a sCT. The final step is the creation of a new treatment plan that exactly matches the anatomy of the day.

Using CBCT for online ART is a dynamic and iterative treatment process. It requires fast image acquisition, quick outlining of all relevant OARs and targets, and rapid plan creation (5). This can be accomplished by embedding automated methods such as auto-contouring and auto-planning within the workflow.

Atlas-based auto-segmentation and CT-to-CBCT deformable propagation of OAR contours makes the deformable transfer of original contours defined on the initial planning CT to daily CBCT rapid and practical (6). Auto-planning systems such as knowledge-based (KB) (7), protocol-based automatic iterative (8) and multicriteria optimization (MCO) (9) allow the planning process to be optimized while also reducing the timing.

ART is intended as a technological improvement offering potential gains in therapeutic outcomes and reduced adverse effects. Plan adaptation is related to anatomical, physiological, and positioning changes observed during therapy. In head and neck cancer patients, such changes can drastically affect the dose distribution and hence the associated toxicities.

Weight loss during radiotherapy for head and neck cancers leads to changes in body contour, fat distribution, and soft tissue thickness, affecting treatment positioning and accuracy. Minor positional shifts in bony structures can also occur due to changes in soft tissue support, affecting patient alignment. Muscle atrophy or changes in muscle mass around the head and neck area also influence patient stability and positioning. Changes in airway and esophagus positions are noted as surrounding tissues respond to treatment, leading to mucositis and dysphagia. Parotid glands often shrink and deform due to their proximity to the radiation field, altering their position and increasing the risk of xerostomia. Similar changes can occur in the salivary glands. Lymph nodes and tumor shrinkage, a common response to radiation, necessitate adjustments to ensure adequate dosing of the remaining tumor mass. Daily ART accounts for these factors and represents a substantial advance in personalized cancer care. However, the implementation of ART presents challenges. Frequent imaging and plan adjustments require sophisticated technology and organization, increasing the complexity and cost of treatment. Leveraging clinical workflow to incorporate adaptive processes without significant delays or interruptions in patient care is mandatory. Full clinical implementation of ART for head and neck cancer is still limited and requires improvement in both technology and practice guidelines before it becomes a new standard.

In this work, an automated workflow for H&N radiotherapy, using the available resources at our facility is analyzed. An Atlas Based Auto Segmentation (ABAS) contouring system is employed for automatic contouring, followed by deformable contour propagation to generate sCT images. Planning is carried out using an MCO *a priori* auto-planning system with a wish-list (WL) for the head and neck region. Subsequently, a proof of concept for an automated workflow leveraging the obtained results is tested for the CBCT online adaptation with conventional linear accelerators. A similar strategy conventionally adopted for MRgRT is replicated for two patients, employing the adapt to shape technique (3). We also evaluate a fluence to position approach with the CBCT. For the CBCTgRT, with a C-Arm linac with a 6 degrees of freedom couch, the fluence to position (FTP) is a couch shift and fluence calculation as opposed to the adapt to position (ATP) in the Unity System where the “virtual couch shift” is implemented. To assess the effectiveness of the workflow, the timing of different phases is considered, the robustness of the WL is evaluated, and the acceptability of a treatment plan is examined applying the two adaptive techniques.

## 2 Materials and methods

**Figure 1:**
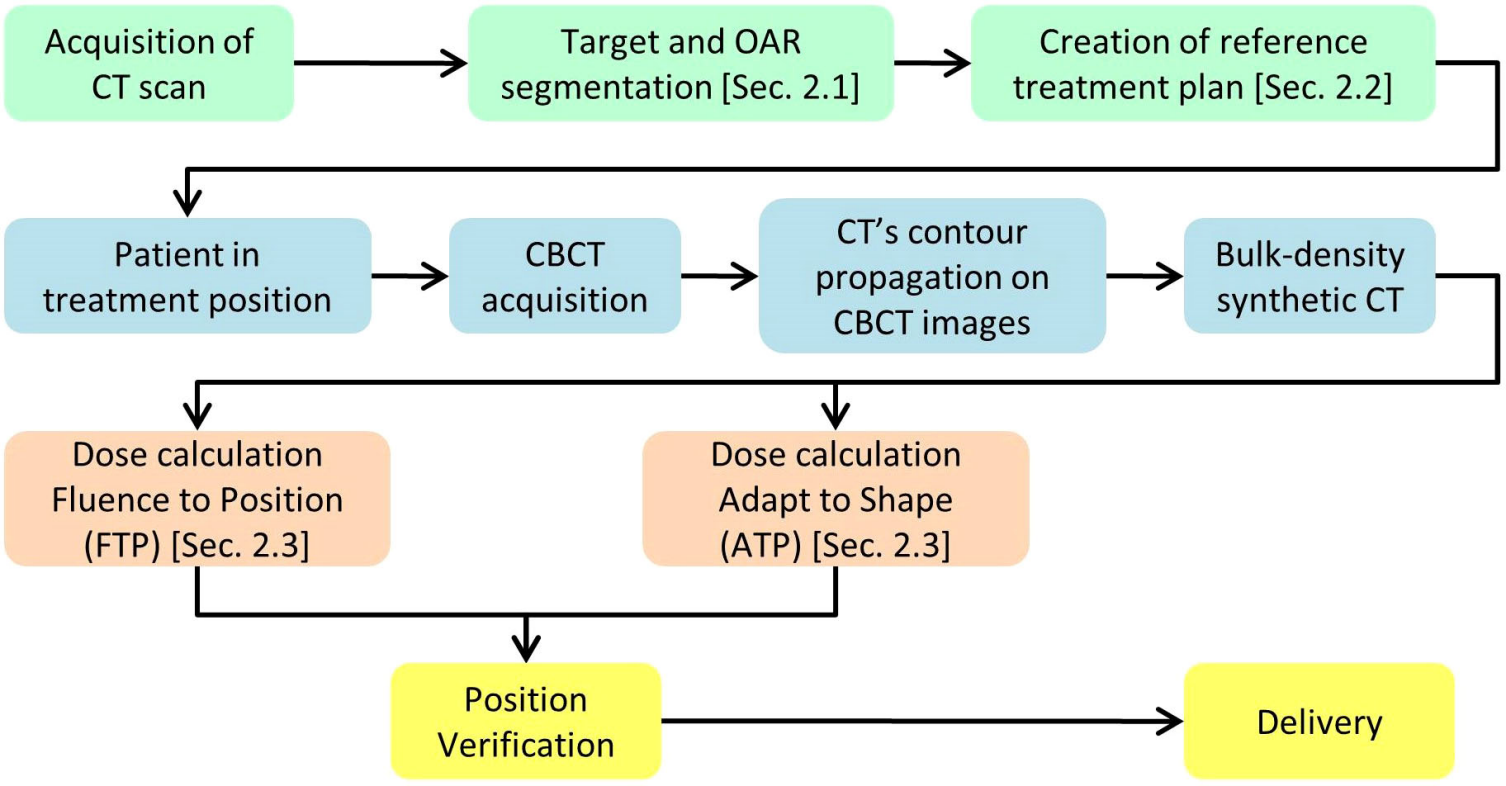
Diagram illustrating the key steps of the workflow.

The workflow starts with the pre-treatment phase, depicted in green in **Figure 1**. This phase starts with a CT simulation, during which the OARs are defined using automatic tools, as described in Section 2.1. Following this, the reference treatment plan is generated using the automatic planning software outlined in Section 2.2. Both the target/OAR definition and plan creation are performed with the involvement of the physician and medical physicist, ensuring accuracy and quality control during the use of the automatic software. The daily treatment workflow begins with the acquisition of a CBCT, which provides a new set of reference images. These images are then imported into the treatment planning software, where the contours from the planning CT are propagated onto the current CBCT by means of the Deformable Image Registration and contours projection. These propagated contours must be reviewed and, if necessary, manually corrected by the physician. Once the CBCT is loaded into the treatment planning system, the Adapt Setup and Force ED options are used to apply bulk density correction to the CBCT images. After generating the synthetic CT (sCT) from the CBCT, clinicians have two options, depicted in orange and described in Section 2.3, for the next step: they can either use the Fluence-to-Position method to verify the dose delivered by the reference plan in the patient’s current position, or they can use the Adapt-to-Shape option to perform an adaptive replan, leveraging the automatic tools described in Section 2.2. Finally, the patient’s position is re-checked, and the selected treatment plan, either the reference plan or an adapted one, is delivered.

### 2.1 Auto-contouring

Although Deep Learning or Atlas Based auto-contouring are used to automatically outline the organs at risk, in daily clinical practice a manual inspection and confirmation is always performed by the Radiation Oncologist prior to allow the optimization phase. ADMIRE^®^ software (ADvanced Medical Imaging Registration Engine, research version 3.37, Elekta AB, Sweden) with random forest (RF) algorithm for the multi atlas-based segmentation on CT images is used. The OARs for the head and neck region considered in this study are: cochleae, mandible bone, larynx, oesophagus, oral cavity, brainstem, optic chiasm, optic nerves, lens, eyes, lachrymal glands, pituitary gland, brain, lips, muscles constrictor, parotids, thyroid gland, trachea, brachial plexus, spinal cord and lungs. Additionally, structures essential to manage CBCT images in the planning process are also introduced. These structures represent different HU layers, divided into external body, air, fat, tissue, and bones. To define these structures for the atlas, the semi-automatic “whole-body” tool available in the MIM^®^ software (MIM Software Inc, version 7.2.8, OH) is exploited. The five HU layers are associated with different voxel’s HU value. The atlas used for the auto-contouring process contains both the previously listed OAR structures and the HU layers.

To assess the accuracy of the HU layer and the OARS structures generated by the auto-contouring software, the volumetric disparities between semi-automatic contours (ground-truth) and the automatic segmentation of nine patients are measured (**Table 1**). The analyzed OARs are divided into large-sized structures (volume>15cc) and small-sized structures (volume ≤ 15cc).

**Table 1:** Patient information for each analysis with cancer diagnosis and staging.

Auto-contouring (9 patients)	Auto-planning (20 patients)	CBCT Adaptive (2 patients)
1 Nasopharynx stage III	4 Nasopharynx stage III 1 Nasopharynx stage IVA	
1 Oropharynx stage I	2 Oropharynx stage I 2 Oropharynx stage IVA	
1 Hypopharynx stage IVB	1 Hypopharynx stage IVB	1 Hypopharynx stage IVB
4 Larynx stage III 1 Larynx stage IVA	3 Larynx stage III 3 Larynx stage IVA	1 Larynx stage IVA
	1 Oral cavity stage III	
1 Parotid stage I	1 Parotid stage IIIB 1 Neck stage IVA	

For the evaluation, the Dice similarity coefficient (DSC) is considered. To perform the geometrical analyses the Golden Rule software (version 1.2, Canis Lupus LLC, Wisconsin, USA) is employed.

### 2.2 Auto-planning

The mCycle algorithm, recently launched under the name of “ElektaONE AutoPlanning”, is used for the a-priori Lexicographic multicriteria plan calculation with a WL for a Simultaneously Integrated Boost (SIB) treatment. mCycle is the Elekta implementation of the iCycle algorithm by Erasmus University and is based on the Lexicographic Approach developed by Sebastian Breedveld (10). The cost functions and the Dose Calculation algorithm are adapted to Monaco. The basis of iCycle is an *a priori* definition of constraints and priority treatment goals. The so-called wish-list can be constructed, goals are optimized sequentially, resulting in a pareto-optimal solution without interactions (11). The overall process is defined as Intelliplan Optimization in the mCycle environment and is summarized in two diagrams included in the **Supplementary Material**. To exploit the auto-planning process, the WL must be tuned and subsequently validated. For the validation of the WL twenty patients are chosen (**Table 1**) with a prescribed dose of 70 Gy to the macroscopic tumor and 56 Gy to the nodes in 35 fractions. For each patient a manual and an automatic plan are calculated using the same calculation settings and sequencing parameters. For the validation, the dosimetric quality of the plans and the planning times are analyzed.

Dosimetric quality is assessed both qualitatively and quantitatively. The qualitative assessment is carried out by a physician, based on a blinded dosimetric comparison between plans. Automatic and manual plans are together presented to the physician using identical layouts of plotted dose distributions and dose volume histograms. The physician assigned to each plan a score from 1 to 5 (1-unacceptable; 2-borderline; 3-sufficient; 4-good; 5-excellent) for target coverage, OARs sparing and plan acceptability. The mean value of the physician’s score and the percentage of automatic plan choices is then analyzed to determine the effectiveness of automated planning over manual planning. The quantitative assessment of the dosimetric quality is carried out by analyzing the dosimetric objectives for PTV coverage and OARs sparing and introducing a plan quality index (PQI) (12, 13). For the dosimetric scores, the median values distribution (with 1st and 3rd quartile) of each constraint for the two planning modalities is observed.

The PQI defines the overall performance of a plan in an operator independent manner. PQIs for PTVs and OARs are first considered independently and then collectively. The generalized formula for the PQI assessment is shown in Equation 1


(1) $$PQI=\sum w\;*\;\frac{D_{x\%}^{goal}-D_{x\%}^{plan}}{D_{x\%}^{plan}}$$

$D_{x\%}$ stands for the dose received by the *x*% of the volume of PTV or OAR, “plan” refers to the dose–volume indexes in the dose plan, “goal” refers to the dose objective and *w* refers to the weighting factor used as function of clinical relevance of the OAR or PTVs. For the PQI calculation of both PTVs, emphasis is placed on four specific points of the DVH to describe its steepness (D_95%_, D_90%_, D_50%_, D_7%_) and on the percentage of the volume covered by the 95% of the prescription dose.

The weight *w* is assessed with the physician on a scale from 1 to 5 to account for the relatively clinical importance assigned by the radiation oncologist team as part of the clinical intent, and it is set equal to 0 for OARs that correspond to the GTVs. Two different templates are defined for the w: one for the NPC cases (wH) and one for the “middle-lower” cases (wL). **Table 2** showed the values used for the calculation. The statistical significance (p < 0.05) of the dosimetric result is evaluated with a signed-rank Wilcoxon test using Python version 3.10.12.

**Table 2:** OARs weights for the calculation of the PQI.

OARs	Objective	wH	wL
Bone_Mandible	D0.03cc	2	2
Brachial_Plex	D0.1cc	2	2
Brain	D0.03cc	3	1
	Dmean	2	1
Brainstem	D0.03cc	5	3
Cochlea	D0.03cc	1	1
	Dmean	4	1
Esophagus	D0.03cc	3	3
Eye	D0.03cc	3	1
Glnd_Lacr	D0.03cc	3	1
	Dmean	2	1
Larynx	D0.03cc	3	4
	Dmean	3	4
Lens	D0.03cc	4	1
Lips	D0.03cc	2	3
OpticChiasm	D0.03cc	5	1
OpticNrv	D0.03cc	5	1
Cavity_Oral	V30Gy	1	1
	Dmean	4	4
Parotid	Dmean	4	5
Musc_Constrict	Dmean	3	4
Pituitary	D0.03cc	4	1
SpinalCord	D0.03cc	5	5

For the two plan modalities, the time needed for the calculation is measured.

The use of automated planning with Lexicographic optimization allows a more uniform set of results that are pretty much independent on the planner experience as they rely on the description of the wish-list as a class solution with the personalization both to intra-patient and inter-patient anatomical variations carried on via the Muli-criteria optimization approach. The wish-lists have been created and tweaked to try to achieve the minimum modulation degree able to assure the achievement of the convergence of the optimization (ATS) throughout all the given fractions and the consistency of the Patient Specific Quality Assurance (PSQA) with a clinically acceptable Gamma Index pass rate. All of the above, in order to assure that results can be applied and replicated to the Head&Neck patient class solution.

### 2.3 CBCT adaptive

Once the auto-planning WL is validated, two patients (**Table 1**) are chosen for a retrospective CBCT adaptive online feasibility analysis: patient A and B. The first seven CBCTs performed on the patients and the first of the following four weeks are selected, for a total of eleven CBCTs. The two modalities of adaptive online investigated are: fluence to position (FTP) and adapt to shape (ATS).

FTP focuses on adjusting the plan isocentre to accommodate daily variations in patient positioning. This mode replicates the current routine clinical practice of treatment delivery where shifts in the x, y, and z coordinates are performed. These shifts are extracted from the R&V (MOSAIQ^®^, Elekta, Sweden) and applied to the plan isocentres. The dose calculation is performed without further plan optimization, using the same patient-specific template previously saved.

The ATS mode takes into consideration not only changes in patient positioning but also anatomical variations, allowing the adaptation of the treatment plan on the shape of the daily patient’s anatomy. For the ATS mode the patient template is calculated and optimized using mCycle without any manual tweaking. The achievements and failures of dose constraints are recorded for the eleven fractions. This assessment involves evaluating the adherence of the adapted plans to the predefined dose constraints established for the OARs and targets. To determine if there are any statistically significant differences between the two adapted plan modalities, the Wilcoxon signed-rank test is performed (p < 0.05) using Python version 3.10.12 in a Colab notebook. Any significant differences between the FTP plans and ATS plans are then further investigated. An analysis of the timing for the different steps required to produce an online adaptive plan is also carried out to assess its clinical applicability.

## 3 Result

### 3.1 Auto-contouring

The DSC of the five HU layer structures, large-sized structures and small-sized structures are reported in **Table 3**. For each layer, the median values and the first (Q1) and third quartile (Q3) are provided.

**Table 3:** Median and interquartile value of the geometric evaluation using DSC index for the HU layer, large-sized and small-sized automatic contours.

Structures	Air	Bones	Tissue	Fat	External	Large	Small
DSC Median	0.66	0.82	0.87	0.80	0.99	0.88	0.67
DSC [Q1;Q3]	[0.60;0.69]	[0.55;0.89]	[0.85;0.88]	[0.63;0.84]	[0.99;1.00]	[0.83;0.92]	[0.52;0.77]

### 3.2 Auto-planning

To assess the quality of the auto-planning system the physician assigned scores and the percentage blind preference choice are provided in **Table 4**.

**Table 4:** Median physician’s scores and percentage of plan preference choices.

	OARs Sparing	Target Coverage	Plan Acceptability	Plan preference choice
Automatic	5.0	4.5	5.0	65%
Manual	4.5	5.0	5.0	35%

The quantitative dosimetric quality evaluation involves the analysis of the dosimetric score cards and the PQI. **Table 5** presents the median values (with Q1 and Q3 in square brackets) of each constraint in both automatic and manual plans. **Table 6** shows the PQI values calculated for each plan. The Wilcoxon test p-value results are provided in **Tables 5** and **6**. Significant p-values are in bold and the best average constraint achieved value is highlighted in gray.

**Table 5:** Constraint median value results for automatic and manual plans, with Q1 and Q3 in square brackets and Wilcoxon test p-value result.

Structure	Constraint	Optional	Mandatory	Automatic	Manual	p
PTV_1_	V_95%_	98%	95%	97.5 [97.0-98.2]	97.6 [96.9-98.9]	0.41
PTV_2_	V_95%_	98%	95%	99.1 [98.8-99.4]	98.9 [98.7-99.6]	0.65
PTV_2-1_	V_95%_	98%	95%	98.7 [98.3-99.1]	98.6 [98.1-99.4]	0.65
PTV_1_	D_50%_		70	70 [70-70]	70 [70-70]	0.41
PTV_1_	D_7%_		73.5	72.1 [71.8-72.2]	71.4 [71.1-71.7]	**<0.01**
PTV_2-1_	D_7%_	66		66.0 [65.1-66.6]	66.2 [65.1-66.7]	**0.02**
Bone_Mandible	D_0.03_ *_cc_*	70	73.5	68.6 [58.3-70.2]	68.5 [55.5-70.5]	0.33
Brachial_Plex_L	D_0.1_ *_cc_*	60	66	60.0 [57.8-64.6]	59.2 [57.4-64.4]	0.97
Brachial_Plex_R	D_0.1_ *_cc_*	60	66	58.9 [57.8-61.4]	57.6 [57.2-60.0]	0.13
Brain	D_0.03_ *_cc_*	72		34.2 [13.6-50.5]	30.4 [9.0-50.1]	**0.02**
Brain	D*_mean_*	30		1.3 [0.6-4.5]	1.5 [0.6-4.2]	0.23
Brainstem	D_0.03_ *_cc_*	54	55	26.7 [11.1-38.3]	22.3 [5.9-31.0]	**0.01**
Cochlea_L	D_0.03_ *_cc_*	60		1.9 [1.3-12.9]	2.2 [1.4-16.7]	**0.02**
Cochlea_L	D*_mean_*	45		1.7 [1.1-10]	2.0 [1.3-15.3]	**0.02**
Cochlea_R	D_0.03_ *_cc_*	60		2.0 [1.2-23]	2.5 [1.4-27.3]	**<0.01**
Cochlea_R	D*_mean_*	45		1.7 [1.1-18.4]	2.1 [1.3-24.5]	**<0.01**
Esophagus	D_0.03_ *_cc_*	45	55	52.4 [49.4-53.8]	53.1 [48.1-56.9]	0.18
Eye_L	D_0.03_ *_cc_*	40	45	1.3 [0.9-3]	1.6 [0.9-4.1]	**<0.01**
Eye_R	D_0.03_ *_cc_*	40	45	1.3 [0.9-3]	1.7 [1.0-4.4]	**<0.01**
Glnd_Lacr_L	D_0.03_ *_cc_*	40		0.7 [0.6-1.9]	0.8 [0.6-2.1]	**0.01**
Glnd_Lacr_L	D*_mean_*	26		0.6 [0.5-1.3]	0.7 [0.5-1.7]	**<0.01**
Glnd_Lacr_R	D_0.03_ *_cc_*	40		0.8 [0.6-1.8]	0.9 [0.6-1.9]	**<0.01**
Glnd_Lacr_R	D*_mean_*	26		0.7 [0.5-1.5]	0.8 [0.5-1.6]	**<0.01**
Larynx	D_0.03_ *_cc_*		66	68.2 [52.9-70.6]	64.9 [54.9-72.1]	0.15
Larynx	D*_mean_*	44	50	24.2 [20.3-58.7]	38.8 [37.1-63.8]	**<0.01**
Lens_L	D_0.03_ *_cc_*	4	10	0.8 [0.6-1.4]	0.9 [0.7-1.9]	**<0.01**
Lens_R	D_0.03_ *_cc_*	4	10	0.8 [0.6-1.7]	1.0 [0.7-2.2]	**<0.01**
Lips	D*_mean_*	30	50	15.4 [8.3-23.5]	25.8 [14.5-30.1]	**<0.01**
OpticChiasm	D_0.03_ *_cc_*		55	1.2 [0.9-5.2]	1.3 [0.9-4.8]	0.94
OpticNrv_L	D_0.03_ *_cc_*		55	1.1 [0.8-4.6]	1.2 [0.9-4.6]	0.28
OpticNrv_R	D_0.03_ *_cc_*		55	1.1 [0.8-4.2]	1.2 [0.9-3.9]	**0.04**
Cavity_Oral	V_30_ *_Gy_*	73%		45.7 [24.9-73.7]	73.2 [42.5-99.6]	**<0.01**
Cavity_Oral	D*_mean_*	30	45	30.8 [20.0-43.1]	41.7 [29.6-50.0]	**<0.01**
Parotid_L	D*_mean_*	20	25	19.8 [17.1-25.2]	21.7 [15.8-26.6]	0.65
Parotid_R	D*_mean_*	20	25	19.4 [16.6-20.9]	19.3 [14.3-24.0]	0.97
Musc_Constrict	D*_mean_*	35	50	51.9 [46.0-55.5]	56.7 [50.0-58.4]	**<0.01**
Pituitary	D_0.03_ *_cc_*		50	1.2 [0.9-7.2]	1.3 [1.0-6.9]	0.46
SpinalCord	D_0.03_ *_cc_*	45	50	29.6 [27.5-30.4]	25.1 [24.2-31.1]	**<0.01**

Dose values are reported in Gy. Optional and Mandatory clinical goals are also reported in the table. Significant p-values are written in bold and the better average constraint achieved value is highlighted in gray.

**Table 6:** PQI median values (OARs, PTVs and total) for automatic and manual plans, with Q1 and Q3 in square brackets and Wilcoxon test p-value result.

	Automatic	Manual	p
PQI_OARs	0.35 [0.24-0.50]	0.32 [0.22-0.46]	**0.01**
PQI_PTVs	0.31 [0.29-0.35]	0.33 [0.31-0.38]	**0.02**
PQI_total	0.68 [0.54-0.83]	0.67 [0.52-0.79]	0.32

Significant p-value are written in bold and the better average constraint achieved value is highlighted in gray.

**Figure 2:**
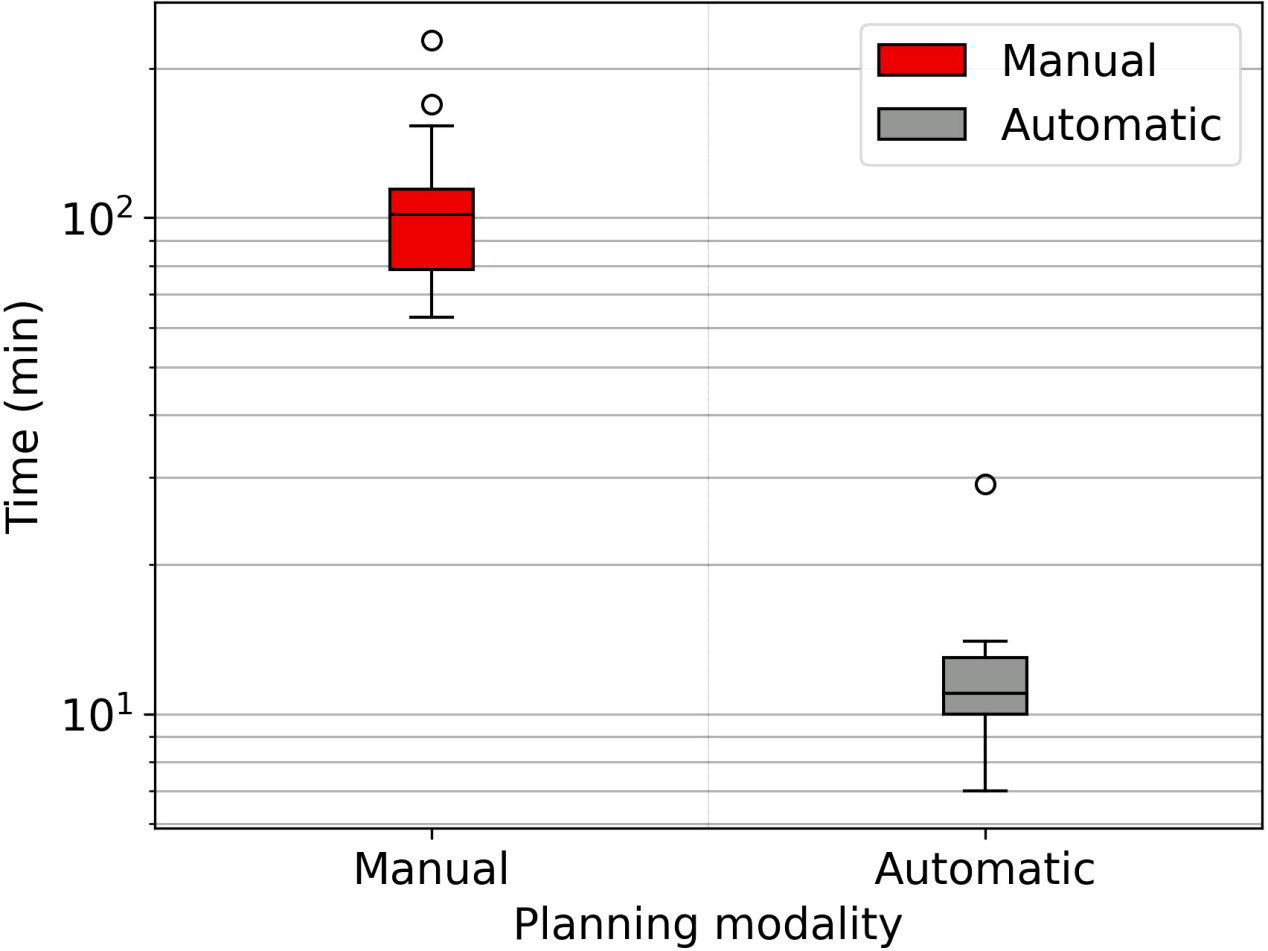
Boxplot showing the time spent for the manual and automatic planning, semilog scale graph.

Planning times are drastically reduced through the auto-planning system. **Figure 2** shows the time difference between the two planning modalities. Automatic planning reaches an average time of eleven minutes, while manual planning is about two hours.

### 3.3 CBCT adaptive

**Figure 3:**
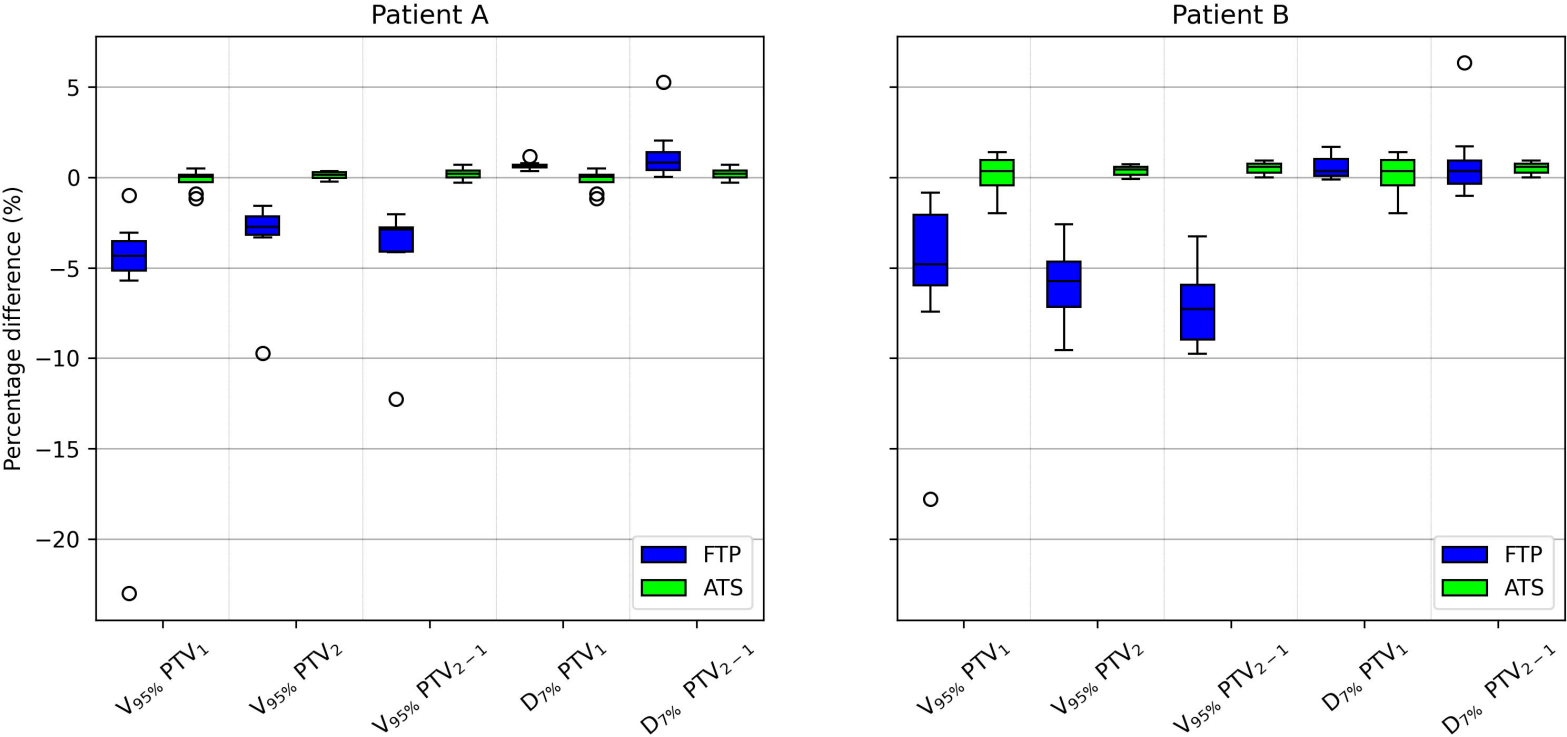
Boxplot showing the PTV_1_, PTV_2_ and PTV_2−1_ percentage difference coverage and PTV_1_ and PTV_2−1_ percentage difference hot-spots between the scheduled plan and the FTP plans and between the scheduled plan and the ATS plans, for patient A and B In the boxplot, the inner line denotes the median value, the box the interquartile range and the whiskers the minimum and maximum value excluding the outliers that are presented as single markers.

**Figure 4:**
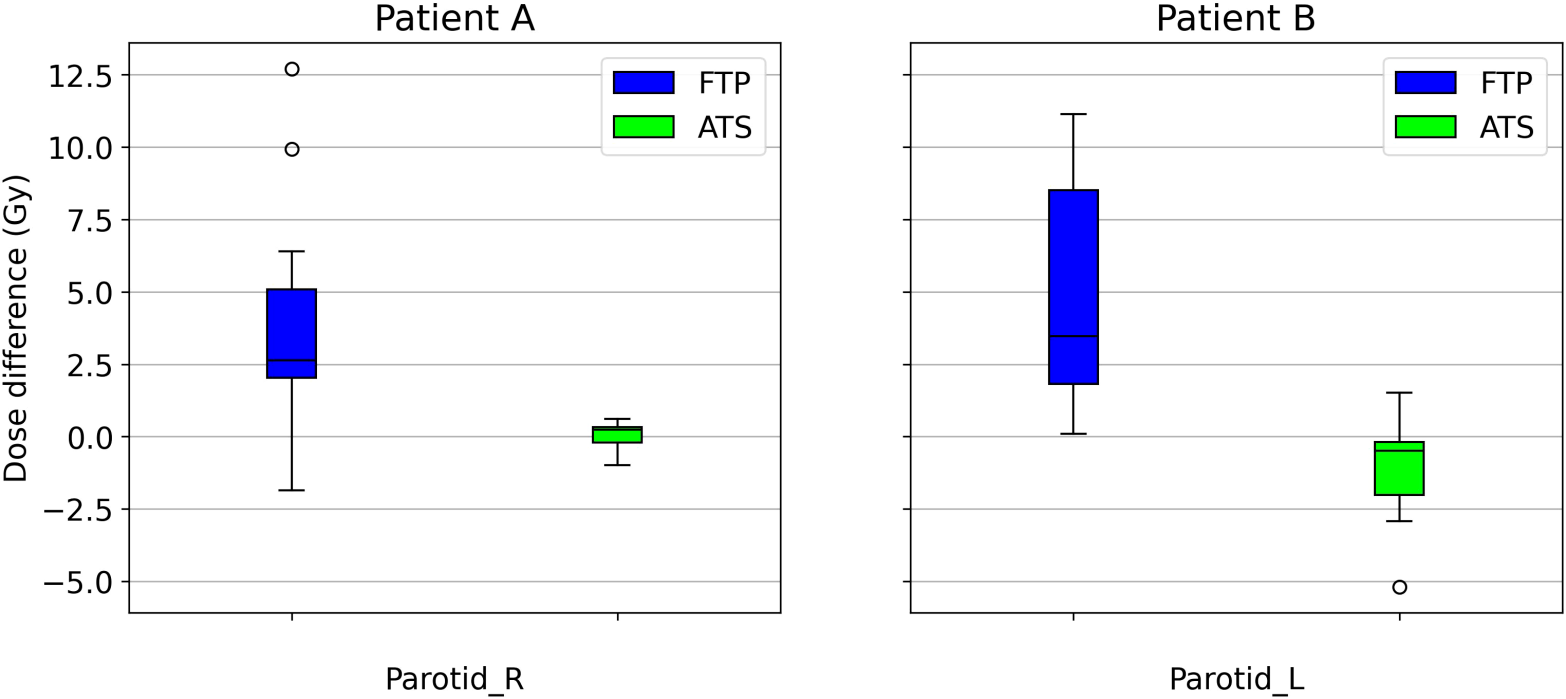
Boxplot showing the mean dose difference between the scheduled plan and the FTP plans and between the scheduled plan and the ATS plans, for patient A and B, only for OARs that are significantly different.

**Figure 5:**
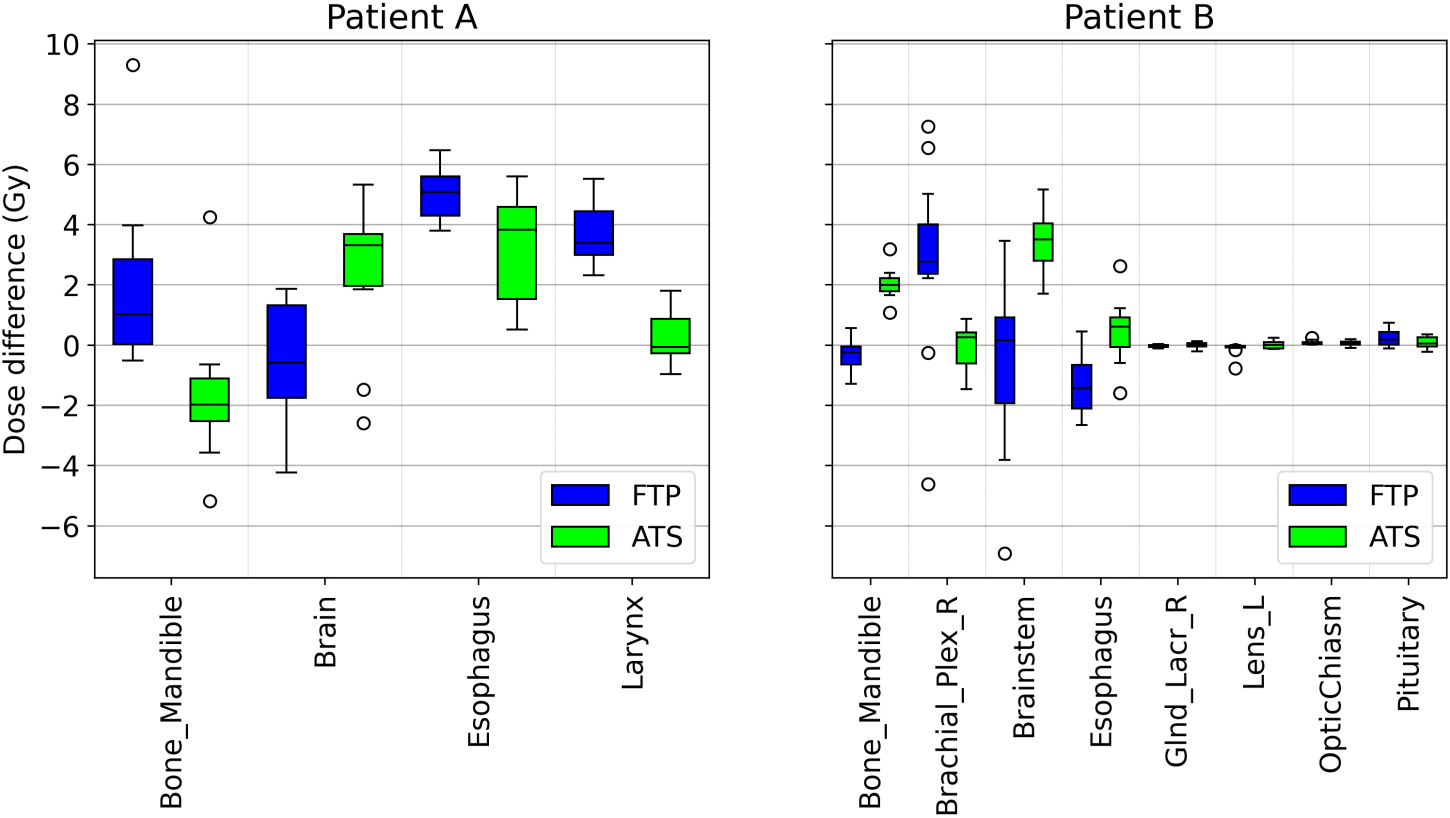
Boxplot showing the max dose difference between the scheduled plan and the FTP plans and between the scheduled plan and the ATS plans, for patient A and B, only for OARs that are significantly different.

The number of passed clinical goal for the two modalities are displayed in **Table 7**, only the constraints that showed a different number between the two modalities are reported. Following the Wilcoxon test to compare the two modes of adaptive planning, the results showed significant differences in the requests for targets and certain OARs. **Figure 3** shows the differences in percentage by coverage and hot-spots of the targets in the two modes for the selected patients. **Figure 4** shows the dose differences for the mean dose constraints, when the results are significantly different in the two modalities. **Figure 5** shows the dose differences for the maximum dose constraints, when results are significantly different in the two modalities. For an evaluation of the clinical feasibility in implementing an adaptive online workflow, the time required for each step of the process is recorded. **Table 8** presents the averages times and their standard deviations obtained for the two adaptive modalities. It includes the estimated times for CBCT acquisitions, both for the pre-treatment scan, currently in use with a standard acquisition protocol, and for the “optional” position verification second scan, performed with a fast protocol. The table also presents the total time, offering an esteem of the time required for the two adaptive modalities.

**Table 7:** Number of passed clinical objectives for the mandatory and [optional] constraints in the two adaptive modes, for patients A and B out of the 11 CBCT fractions analysed.

Patient A			
Structure	Constraint	FTP pass Mandatory [Optional]	ATS pass Mandatory [Optional]
PTV_1_	V_95%_	1 [0]	11 [1]
PTV_2_	V_95%_	10 [0]	11 [11]
PTV_2-1_	V_95%_	7 [0]	11 [11]
PTV1	D_50%_	10	11
Brachial_Plex_L	D_0.1_ *_cc_*	10 [0]	11 [0]
Esophagus	D_0.03_ *_cc_*	0 [0]	2 [0]
Cavity_Oral	D*_mean_*	11 [10]	11 [11]
Parotid_L	D*_mean_*	10 [7]	11 [7]
Parotid_R	D*_mean_*	8 [2]	11 [11]

Patient B			
Structure	Constraint	FTP pass Mandatory [Optional]	ATS pass Mandatory [Optional]
PTV_1_	V_95%_	3 [0]	11 [11]
PTV_2_	V_95%_	2 [0]	11 [11]
PTV_2-1_	V_95%_	0 [0]	11 [11]
PTV_1_	D_50%_	8	11
PTV1	D_7%_	10	11
Bone_Mandible	D_0.03_ *_cc_*	11 [10]	11 [0]
Brachial_Plex_ L	D_0.1_ *_cc_*	11 [8]	11 [10]
BrachialPlex_R	D_0.1_ *_cc_*	2 [0]	11 [0]
Cavity_Oral	D*_mean_*	11 [2]	11 [4]
Parotid_L	D*_mean_*	6 [0]	11 [7]
Parotid_R	D*_mean_*	11 [6]	11 [11]

**Table 8:** Average times ± SD (when available) for each step and total time of the FTP and ATS workflow for both patients.

		Pretreatment CBCT (mm:ss)	Adapt anatomy tool-contour (mm:ss)	Replanning time (mm:ss)	Position verification (mm:ss)	Dose delivery (mm:ss)	Total time (min)
Patient A	FTP	01:10	01:06 ± 00:04	01:32 ± 00:02	00:40	04:10	≈9
	ATS			11:06 ± 00:44			≈18
Patient B	FTP	01:10	01:05 ± 00:04	01:37 ± 00:03	00:40	04:27	≈9
	ATS			13:33 ± 01:05			≈21

## 4 Discussion

In this study, we demonstrated the feasibility of implementing an adaptive online treatment without dedicated systems, but leveraging a combination of available automated software. Our project is the first to leverage this combination of automated software (auto-contouring, auto-planning, sCT creation) on conventional accelerators in an Elekta environment.

### 4.1 Auto-contouring

The software used for the OARs auto-contouring was already validated for clinical application and our results are in agreement with the literature (14, 15). However, no results for the auto-contouring of HU layers are obtained on CT images. Instead, DSC values from MRI images are available (16, 17). Our results for bones, tissue, fat and external reach a DSC median value of over 0.8, while for air the DSC median value is 0.66. The external and bones results are comparable to those reported by Guerreiro (16), and for air, fat, and tissue to those reported by Hsu (17). The auto-contouring process shows promising results and strongly facilitates the creation of sCTs necessary for the following adaptive process.

### 4.2 Auto-planning

The WL definition for the mCycle auto-planning involves a precise iterative tuning process, which may take several days. Familiarity with planning in Monaco certainly accelerates the initial steps of this process and the time needed to develop a robust WL is influenced by the level of detail in the defined protocol. When the protocol is more explicit and detailed, mCycle finds easier to get the expected results efficiently. In our case, we took this opportunity to update the clinical constraints commonly used for H&N cases. After the physician’s review of all constraints, the first WL is defined and it is then tested and modified until an optimal solution is obtained for all cases. In the qualitative and quantitative dosimetric evaluation of the automatic treatment plans, favorable outcomes have been achieved. The physician deems all plans clinically acceptable, and in the majority of cases (65%), the automatic plan is chosen over the manual one, as shown in **Table 4**. The automatic plans are preferred by the physician due to their optimal compromise between PTVs coverage and OARs sparing. This finding aligns with the data presented in **Table 6**. The PQI_OARs value is higher for the automatic plans, indicating its ability to achieve more sparing of the critical organs. This result suggests that the automatic plans offer greater overall sparing of the OARs at the cost of slightly reducing coverage of the PTVs. However **Table 5** shows that the requests for PTVs coverage are always well fulfilled in the automatic plans. mCycle auto-planning, enables greater OARs sparing while meeting the target coverage requirements set in the WL. This outcome is consistent with the literature findings on *a priori* MCO auto-planning (9, 10, 13). Looking at the achieved result, we noticed that the constraints of constrictor muscles and larynx are within those modified in the upgrade of our hospital’s protocol. Therefore, from the standpoint of the manual planner, there has been an initial response to address these new requirements, and over time better results could be reached. This challenge is not encountered in the investigated auto-planning system, where modifying a clinical protocol involves the adjustment of a parameter in the WL that is automatically optimized during the calculation. The WL approach allows for easy modification as needed, making it highly adaptable to meet specific new requirements or clinical needs. This cannot be asserted for all auto-planning systems, for example, the KB automatic plans rely heavily on the manual plans and the protocol used up until that point (7). The mCycle auto-planning allows real-time adjustments, supporting, rather than replacing, the role of the medical physics expert to achieve optimized results in a short time. The time required for planning is shown in **Figure 2**, the automatic modality significantly reduces the planning time from about 2 hours in manual mode to about 10 minutes in the automatic mode. The time-saving advantage of auto-planning over manual planning is also highlighted in different studies. Focusing on those related to head and neck cases, one study compares the automatic planning times of *a posteriori* MCO, of a protocol-based automatic iterative approach and of a KB methods, resulting in 31 ± 4 minutes, 83 ± 10 minutes, and 27 ± 4 minutes, respectively (18). In other studies focused on KB, planning times of around 30 minutes (19) and 60 minutes (20) are obtained. For the protocol-based automatic iterative approach, planning times exceeding one hour are also reported (20, 21). For *a priori* MCO, one study reports calculation times of around 60 minutes (13).

In our case, the time required for manual planning aligns with the above-cited publications (around 2 hours per patient’s plan), while for automatic planning, there is a significant reduction not only compared to all other auto-planning systems, but also when using the same auto-planning system with a different WL. Reducing planning time through auto-planning systems (**Figure 2**) allows adaptive online sessions to be implemented.

### 4.3 CBCT adaptive

The starting point is the audit of the feasibility of all steps in the workflow, analyzing the difficulties and proposing potential solutions for future clinical applications. The auto-contouring of different layers (anatomical structures and HU layers) facilitates the creation of sCTs, which are made using bulk density override. The quality of CT-adapted contours on daily CBCTs is validated, and reported in literature (6). The time required for the physician to check these contours is not considered in this project. However, the contours propagated are the auto-contoured ones which have already been reviewed and corrected by the physician, thereby errors such as missing or major inaccurate contours that typically require extensive revision, are reduced.

The analysis of the two adaptive modalities “Fluence To Position” (FTP) and “Adapt To Shape” (ATS) reveals a better correspondence of the ATS plans with the reference CT plans compared to the FTP ones. The number of passed clinical goal, reported in **Table 7**, shows that FTP plans fail to meet the target coverage and also some OARs clinical constraints.

Among the constraints used, the same ones shown in **Table 5**, there are significant p-values for target coverage, hot-spots and some OARs objectives. The percentage difference for target coverage and hot-spots between the value obtained in each fraction and the reference CT is shown in **Figure 3** for both modalities. Moreover, the dose difference with a significant p-value of the mean and max dose objectives for patient A and B are reported respectively in **Figures 4** and **5**. From the PTV graphs, the inadequate coverage of targets in FTP modality is further highlighted, whereas for ATS, it is optimized in each fraction. Regarding the parotids, which in both patients are close to the PTV, an increase in the mean dose objective is observed in FTP modality, while for ATS, it remains constant. For maximum dose values, both increases and reductions are observed in both modalities. The variation in maximum dose delivered to OARs did not affect the acceptability of the plans. However, the low target coverage achieved in FTP mode negatively impacts their acceptability.

Analysis of the two adaptive modes, FTP and ATS, shows that re-optimizing the plan based on the images acquired at the beginning of the treatment session yields superior results, particularly in enhancing PTV coverage and optimizing sparing of OARs, especially those in close proximity to the target.

Adaptive radiotherapy requires the patient to maintain the treatment position at the linac until the adaptive process is completed. This aspect makes it unsuitable for manual contouring-planning methods. Auto-planning enables the calculation of the treatment plan based on the daily anatomy while maintaining the time limited. For the two analyzed patients, the ATS re-planning time is less than 14 minutes (**Table 8**), which is also supported by the automatic calculation time results in **Figure 2**. The estimated workflow total time for online ATS treatment is approximately 20 minutes, which is aligned with data reported in the literature (5, 22). Furthermore, these processing times can be reduced by leveraging more powerful computing systems (e.g. Graphic Processing Units, GPUs). The time required for the clinician to check the contours quality must also be considered in the evaluation of the timing, but in our case this is not assessed. The use of CBCT images for adaptive planning is a topic that has been explored for several years now, exploiting both the use of average structure density override (23, 24), the use of deformed CT images on CBCT (25, 26), and the establishment of Hounsfield numbers versus densities curves (27). The innovation lies in the online implementation of this technique. Currently, this topic is gaining considerable interest, especially due to the introduction of systems that facilitate, automate, and expedite the various steps involved in this process [e.g. ETHOS, Varian Medical Systems (22, 28, 29)].

## 5 Conclusion

This project has undertaken an in-depth analysis of an automated workflow for H&N radiotherapy, using the available resources at our facility.

The auto-contouring approach facilitates the creation of sCTs necessary for the adaptive process. The mCycle auto-planning significantly reduces planning time while maintaining or improving clinical acceptability. In the feasibility study of online adaptive radiotherapy, the ATS mode, which optimizes treatment based on daily anatomy, demonstrates superior outcomes compared to the FTP mode.

Time is a pivotal factor in online adaptive approaches, as all must be managed within the context of a single treatment session. ATS demonstrates efficiency in terms of time, with an estimated total treatment time of about 20 minutes. This outcome marks a preliminary step toward clinical implementation.

The analysis of adaptive methods is focused on feasibility and preliminary evaluation, but the potential of this automated workflow to improve the clinical practice and the patient outcomes remains significant. Further investigations into online adaptive approaches, including retrospective clinical studies with a larger cohort and prospective studies, will contribute to unveiling the complete spectrum of benefits and limitations, particularly regarding the choice between adaptation strategies for each fraction, which remains a critical consideration for future optimization.

This study contributes to the advancement of automatic and adaptive radiotherapy, demonstrating the potential of an automated workflow in challenging cases, such as H&N treatments. The successful validation of auto-contouring and auto-planning software, combined with preliminary findings on online adaptive, underscores the significance of exploiting technology to optimize treatment and improve care for radiotherapy patients.

## Data Availability

The raw data supporting the conclusions of this article will be made available by the authors, without undue reservation.
