# Supplementary material for: Proof of concept of fully automated adaptive workflow for head and neck radiotherapy treatments with a conventional linear accelerator
Source: Front Oncol. 2025 Jan 23;15:1382537. doi: 10.3389/fonc.2025.1382537 (PMC11799547; doi:10.3389/fonc.2025.1382537)

Supplementary Material

1 SUPPLEMENTARY TABLES AND FIGURES

1.1 Figures

Figure S1. Diagram of the Phase 1 Intelliplan Optimization process in the mCycle environment.

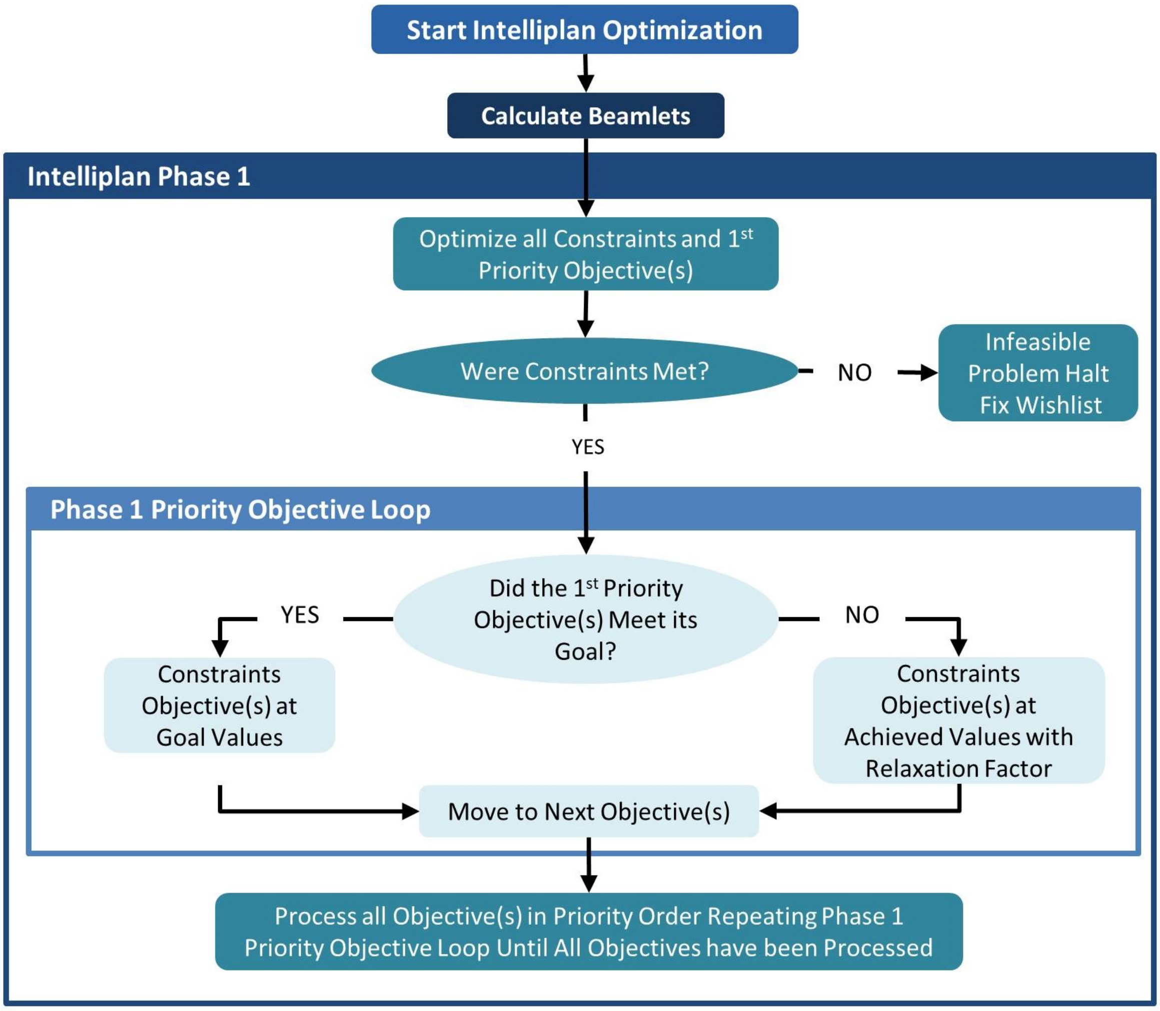

Figure S2. Diagram of the Phase 2 Intelliplan Optimization process in the mCycle environment.

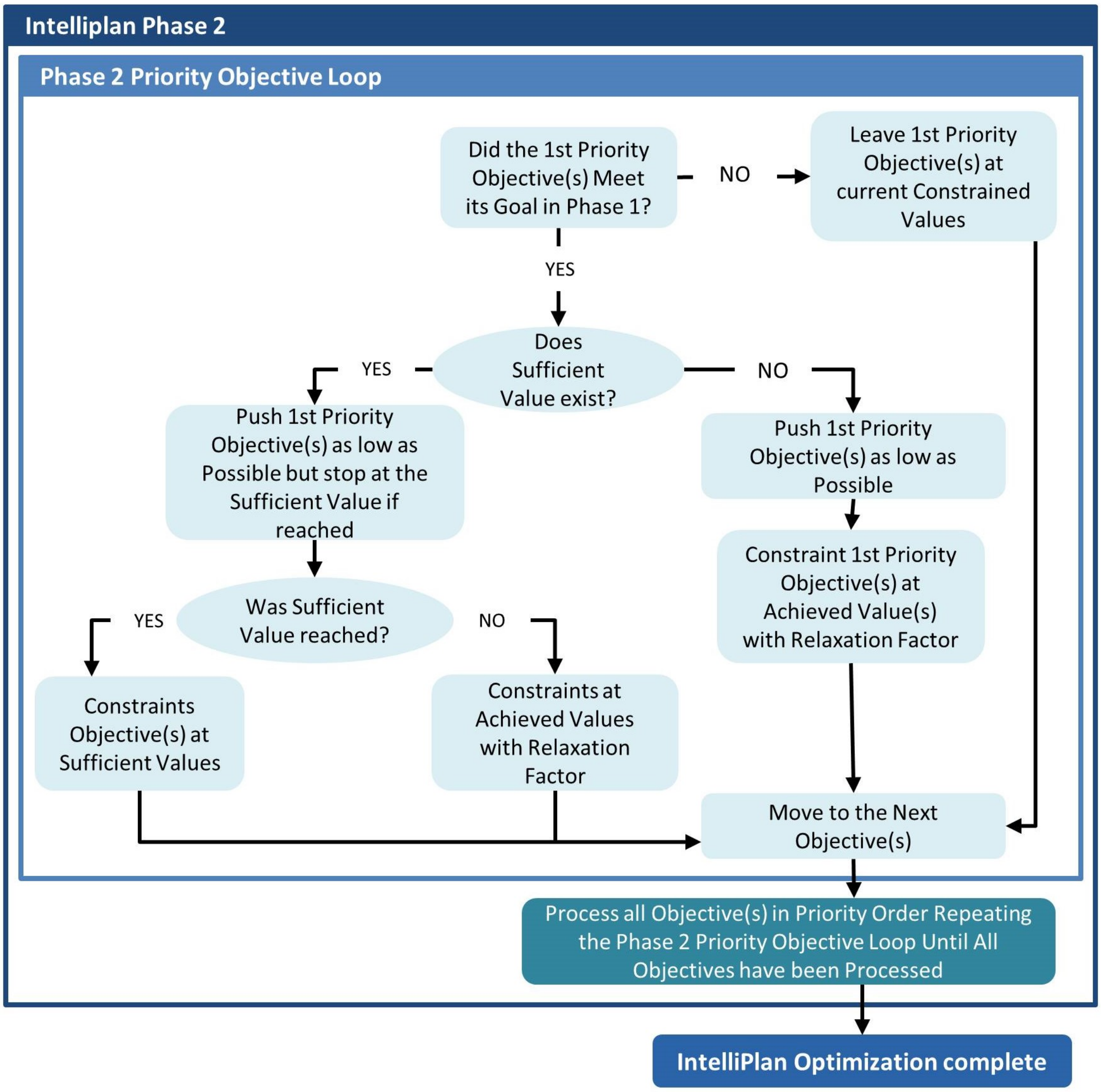

Supplement: Supplementary file 1 [file Image1.pdf]
